# Axial Compressive Properties of Fiber-Reinforced Polymer–High-Water Material–Polyvinyl Chloride Plastic Double-Wall Hollow Column

**DOI:** 10.3390/polym15163351

**Published:** 2023-08-09

**Authors:** Haojie Yin, Hui Chen, Hongqian Hu, Lei Zhang, Huwei Li

**Affiliations:** 1 School of Geology and Mining Engineering, Xinjiang University, Urumqi 830046, China; yinhaojie@stu.xju.edu.cn (H.Y.);; 2 Key Laboratory of Environmental Protection Mining for Minerals Resources, Education Department of Xinjiang Uygur Autonomous Region, Xinjiang University, Urumqi 830046, China; 3 School of Resources and Safety Engineering, Central South University, Changsha 410083, China

**Keywords:** underground mine, composite support, high-water, fast-setting material, polyvinyl chloride plastic pipe, fiber-reinforced polymer

## Abstract

To further enrich the side-filling structure system of goaf-retaining roadways and explore the compression reaction mechanism of the composite in the support environment of underground mine roadways, this paper introduces a double-wall hollow composite pier structure (FPRSC structure) that is composed of the fiber-reinforced polymer (FRP) composite and polyvinyl chloride plastic (PVC) as restraint materials and the infill material featured with a high water-to-powder ratio. A total of 16 circular specimens with a diameter and height of 100 mm were tested to explore the axial performance of the combined support structure. The main control variables in the present research included the water-to-cement ratio of the high-water material (e.g., 2:1, 3:1, and 4:1), the thickness of the FRP pipe (i.e., 6 mm and 3 mm), the inner diameter of the PVC pipe (i.e., 29 mm and 22 mm), as well as the thickness of the PVC pipe (1.5 mm and 5 mm). Test results showed that the high-water material was under triaxial stress due to the double-wall tube binding, and the bearing capacity of the composite was higher than that of the single material. Meanwhile, the FPRSC structure exhibited obvious strain-hardening characteristics when the infill material is under the combined constraints of double-wall hollow tubes. Moreover, the ratio of PVC-c, FRP-A, and high-water material with a water–cement ratio of 3:1 shows the best axial mechanical properties. The new composite pier structure with high toughness and strength has wide application prospects in the field of goaf retention in deep underground mines.

## 1. Introduction

Gob-side entry retaining technology is an important development direction of coal mining. A large number of scholars have conducted in-depth research on this subject [1,2]. Currently, the main forms of retaining lanes along the gob include concrete-filled steel tubes, high-water material pier columns, concrete walls, hydraulic support, etc. [3,4,5,6]. Fiber-reinforced polymer (FRP) composites have been widely used in civil engineering attributed to their good mechanical properties [7,8,9], the advantages of which have been well demonstrated. In recent years, some scholars have proposed new support structures incorporating FRP composites [10]. These novel structural forms are FRP-constrained high-water-material pier columns [11,12], FRP-constrained high-water material with coal gangue columns [13], PVC-constrained high-water-material columns [14], and other structures. Based on previous research, a double-wall structural form, whereby the exterior container is made of an FRP-PVC tube and the infill material is made of high-water material, is proposed in the present research.

Being an innovative composite material, FRP has excellent strength and toughness associated with low weight and good wear. FRP is widely used in engineering structure reinforcement, and detailed research have been previously carried out both at home and around the world [15,16]. Wang et al. [17] conducted impact resistance tests on 24 FRP–concrete–steel tube composite column specimens and found that the composite column specimens obtained good ductility and good impact resistance. Xu et al. [18] applied a theoretical analysis and experimental investigation to explore the influencing factors of CFRP-confined concrete and found that the failure of concrete columns was caused by the redistribution of CFRP-confined stress and structural weakening. By comparing the two sets of tests of FRP tube–concrete/steel tube composite column and GFRP tube–high-strength concrete–high-strength steel tube, Yu et al. [19] revealed that the ratio of steel tube diameter to thickness had a greater influence on the bearing capacity of the FRP–concrete–steel tube composite column, but it had a smaller influence on the bearing capacity of GFRP tube–high-strength concrete–high-strength steel tube composite column. Li et al. [20] investigated the mechanical properties of FRP-confined concrete-filled steel tube (CFST) long columns and proposed the expression of stability coefficient suitable for specimens. 

As reported by Watanable et al. [21], CFRP and AFRP were adopted as the confining material to restrain the lateral dilation of infilled concrete. Matthys et al. [22] tested four FRP-confined concrete under axial compression loading, where the diameter and height of tested specimens are 150 mm and 300 mm, respectively. Teng et al. [23,24,25] introduced an FRP–concrete/steel double-wall hollow composite column, both the axial compressive and flexural mechanical properties of which were systematically investigated. Saingam et al. [26] investigated the axial bearing performance of concrete confined by HFRP. Different from the plain concrete core, the ultimate compressive strength and strain of FRP-confined concrete increased by 272% and 457%, respectively. In terms of loading methods, the research on composite specimens of CFST constrained by FRP is not limited to axial monotone loading. Compared with monotone loading, composite specimens under cyclic loading showed increased compressive strength and strain [27]. In addition, the binding form of FRP has a great impact on the overall strength of the specimen. Previous research have clarified that a hooped ring binding with appropriate spacing can save the amount of FRP material when the strength of combined specimen can be ensured [28].

**Figure 1:**
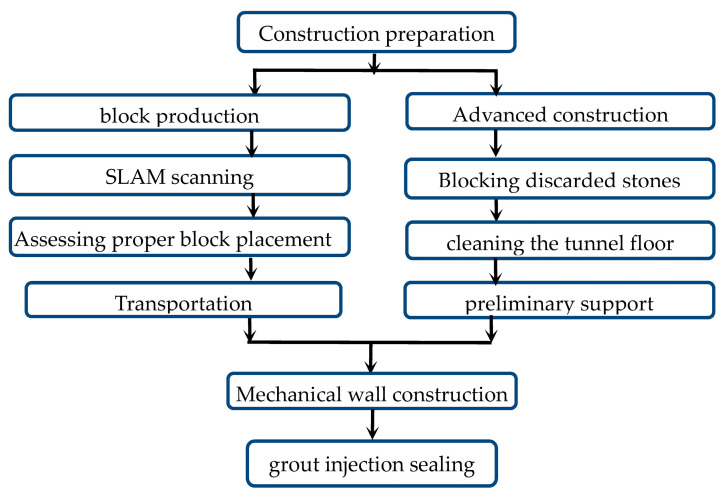
Construction flow chart.

Against aforementioned research, a new support structural form with a prefabricated double-wall hollow column with adjustable height is proposed in the present research. During the construction of the structural form, the double-wall hollow columns will be prepared on the ground in advance and then transported underground for assembly or direct use. Note that complicated procedures of traditional support forms underground are thus not required. The height adjustment mechanism can be fine-tuned according to the actual installation height of the roadway, and the operation is convenient and fast, which solves the problem that the height of the supporting column cannot be accurately determined due to the variation in the rock mass level in the roadway. Meanwhile, the FRP tube high-water coagulation material/PVC-tube double-wall hollow pier column shared the advantages of a good corrosion resistance, superior deformation resistance, slow failure process, and high bearing capacity after the initial failure of the assembly. The specific construction methods are listed below for reference: (1) the double-wall hollow-column assembly block was prefabricated; (2) the assembled block was built with special equipment for retaining lanes and filling along the goaf; (3) support columns with adjustable heights were used to connect the roof, and gunite pipes were used to spray the constructed wall, which realizes the mechanization of goal-side lane retention filling and reduces the cost of gob-side lane retention filling. The specific construction process can be seen in Figure 1.

The bearing performance of the backfill beside the double-wall hollow column roadway is the basis for controlling the movement of overburden in the later stage. Therefore, this paper intends to study the bearing deformation mechanism of the backfill beside the double-wall hollow column roadway through mechanical tests, and analyze influence factors such as the internal and external pipe walls, water-to-cement ratio of high-water material, as well as the hollow ratio. The ultimate bearing capacity and mechanical bearing characteristics of the double-wall hollow column under the axial compression loading are investigated. Moreover, the technical parameters of the filling body are discussed.

Different from traditional standing supports applied for the gob-side entry retaining technology, the proposed structural form obtains some other advantages:

1. A double-wall bound hollow column is designed by combining the low-cost PVC pipe, FRP pipe and high-water material. The support body is of low cost, good corrosion resistance, and superior compressive performance. Moreover, it may sustain high strength after failure, and can ensure the slow deformation of the target rock mass under unidirectional compression conditions. In addition, the novel support greatly improves the overall safety of underground mines.
2. The axial compressive properties of the composite columns are closely related to the exterior container and the infill material. As per previous research on FRP composite column, this novel column may have a better axial mechanical properties compared with that of a single supporting body. Most importantly, the optimal composite material parameters will result in other advantages when it is used in underground mines with variable geological conditions.

## 2. Materials and Methods

### 2.1. Test Sample

**Figure 2:**
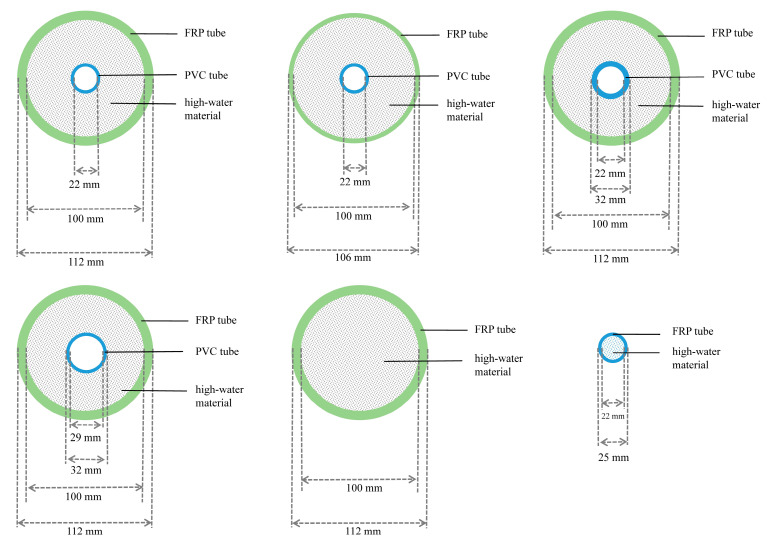
Schematic diagram of the basic combination form and size of the specimen.

A total of 16 composite samples were prepared at Xinjiang University. According to the idea of controlling a single variable, the composite specimens of 6 mm thick FRP tube, 22 mm thickness, 1.5 mm PVC tube, and a high-water material with a water-to-cement ratio of 3:1 were used as the control group. Four control experimental groups were further designed to study the effects of different material parameters on the axial pressure properties of the combined specimens. Among the 16 combined samples, 6 high-water materials had different water–cement ratios (i.e., 2:1, 3:1, and 4:1) to explore the axial mechanical properties of samples with different water-to-cement ratios. Two PVC pipes shared the same thickness (i.e., 1.5 mm) and different inner diameters (i.e., 29 mm), and two PVC pipes are of the same inner diameter (i.e., 22 mm) and different wall thicknesses (i.e., 5 mm) to explore the axial mechanical properties of different PVC pipe parameters. Two FRP tubes have the same inner diameter (i.e., 100 mm) and different thicknesses (i.e., 3 mm) to investigate the influence mechanism of FRP tube thickness on the axial compression properties. Each group of samples was compared with the basic experimental group to achieve the principle of controlling a single variable. In addition, 2 FRP tubes and 2 PVC tubes were fully loaded with high-water materials to explore the influence of hollow conditions on the combined specimens. It should be noted that the height of all specimens is 100 mm and the uniform curing time is 3 days. More detailed information about the experimental specimens can be seen from Table 1, and the specific combination form and size of specimens are shown in Figure 2.

To make the experiment more rigorous, axial pressure tests on a single FRP tube and PVC tube were also carried out to compare the axial mechanical properties and deformation characteristics of the combined specimens. Moreover, 15 cylindrical specimens of plain high-water material with a height of 100 mm and a diameter of 50 mm were prepared, including 3 high-water material ratios with water-to-cement ratios of 2:1, 3:1, and 4:1, respectively. Five identical specimens were set for each ratio to increase the reliability of the data. More detailed experimental information is listed in Table 1. 

In order to facilitate the reference, each sample is named, in which the first uppercase English letter represents the FRP tube with different parameters. In detail, letter A represents FRP tubes with a thickness of 6 mm, letter B represents FRP tubes with a thickness of 3 mm, the second lowercase letter in the specimen label represents PVC pipes with different parameters. Letters a, b, and c represent the thickness of 1.5 mm. The PVC pipe with an inner diameter of 22 mm, thickness of 1.5 mm, an inner diameter of 29 mm, a thickness of 5 mm, and an inner diameter of 22 mm are followed by the hyphen number, then the first Arabic number, which represents the ratio of different high-water materials, and finally the Arabic numbers 2, 3, and 4, which represent the water–cement ratio of 2:1, 3:1, and 4:1, respectively. Moreover, the last Roman numeral is adopted to distinguish the same specimen from the same type of specimen. Taking the specimen Aa-3-1 for example, it is a composite specimen with a 6 mm thick FRP tube and a 22 mm inner diameter PVC tube with a thickness of 1.5 mm, and a water-to-cement ratio of infill material of 3:1.

### 2.2. Experimental Introduction

#### 2.2.1. Specimen Preparation Process

**Figure 3:**
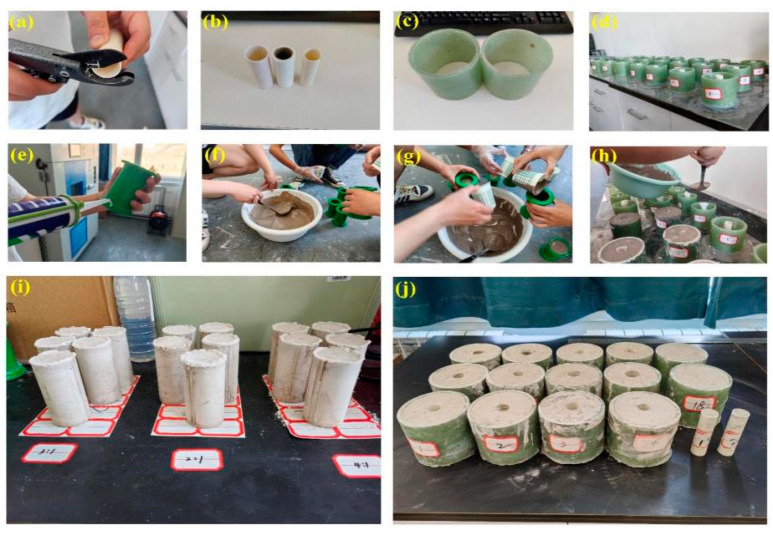
Specimen preparation process: (**a**) cutting PVC pipe; (**b**) molding PVC pipe; (**c**) forming FRP tubes; (**d**) mixing material; (**e**) die sealing; (**f**) preparation of high-water materials; (**g**) single high-water-material sample placement; (**h**) composite specimen placement; (**i**) single high-water-material sample display; (**j**) assembly of sample display.

The following process was performed to complete the production of composite specimens: (1) purchasing the PVC pipe, the chlorine content of which is more than 66%; (2) selecting FRP pipes of two thicknesses produced from the same batch; (3) smoothing PVC pipe and FRP pipe by the acrylic plate, and further adopting the glass glue to seal the bottom of the combined pipe to prevent water leakage; (4) mixing high-water material with different ratios—note that it is necessary to stir A and B materials with the requested water within three minutes and then mixing A and B materials for two minutes—and (5) after the high-water material has a certain initial setting strength, the specimen will be molded, and the axial mechanics experiment is carried out after three days. The specific specimen preparation process is shown in Figure 3. Note that a total of 46 test samples were prepared, including 12 FRP tube/PVC tube/high-water-material assembly samples, 2 FRP tube/high-water-material assembly samples, 2 PVC tube/high-water-material combination samples, 9 single PVC pipe material samples, 6 single PVC pipe material samples, and 15 single high-water-material samples with different water–cement ratios. For each group, there are 2 identical samples. For the final test result, the average value of the same sample is taken and the standard deviation is calculated.

#### 2.2.2. Specimen Preparation Process

The uniaxial test equipment is produced by MDS Systems Company in Edinburgh, MN, USA. The model is LPS.605, series No. 1213562, and the maximum loading pressure is 600 kN. The compression machine was driven by displacement control and a constant loading speed of 0.06 mm/s was adopted. According to the characteristics of the strong deformation ability of the experimental material, the loading was automatically stopped when the loading displacement was set in the loading system to reach 50 mm, and the loading of a single specimen was completed.

## 3. Results

### 3.1. High-Water Fast Setting Material

The high-water–cement material was produced by Xucheng Mining Technology Development Co., Ltd. in Xuzhou City, Jiangsu Province, China. It is of a high-alumina filling material with a water volume ratio of 95–97%. The main raw components of the high-water material include material A (bauxite, gypsum), material AA (composite superplasticizer), material B (gypsum), material BB (composite accelerator), etc. In detail, material A and material B form separate slurries when mixed with water. These two single slurries are then mixed in appropriate proportions and are uniformly stirred to form a consolidated mass with certain strength within a specific time period. In practical on-site applications, mining personnel establish high-water–cement material filling pump stations on the ground. The slurry system automatically prepares slurries A and B separately. The premixed slurries are then transported to the filling working face near the goaf through a pipeline system in proportion. After mixing, the combined slurry is injected into the goaf through the filling pipeline to complete the filling process. Due to the material’s characteristic of rapid setting within a short time for pumping through pipes, a certain degree of fluidity is required. Through indoor experiments, it was found that the material ratio of 1:1 and 1:1.5 for the high-water–cement material resulted in an excessively fast solidification, causing the high-water material slurry to lose its fluidity and impede pumping. Based on multiple tests, it was determined that the high-water–cement material ratios of 2:1, 3:1, and 4:1 exhibit good fluidity after mixing. Thus, the water-to-cement ratios of 2:1, 3:1, and 4:1 for the high-water–cement material were chosen for this experiment.

**Figure 4:**
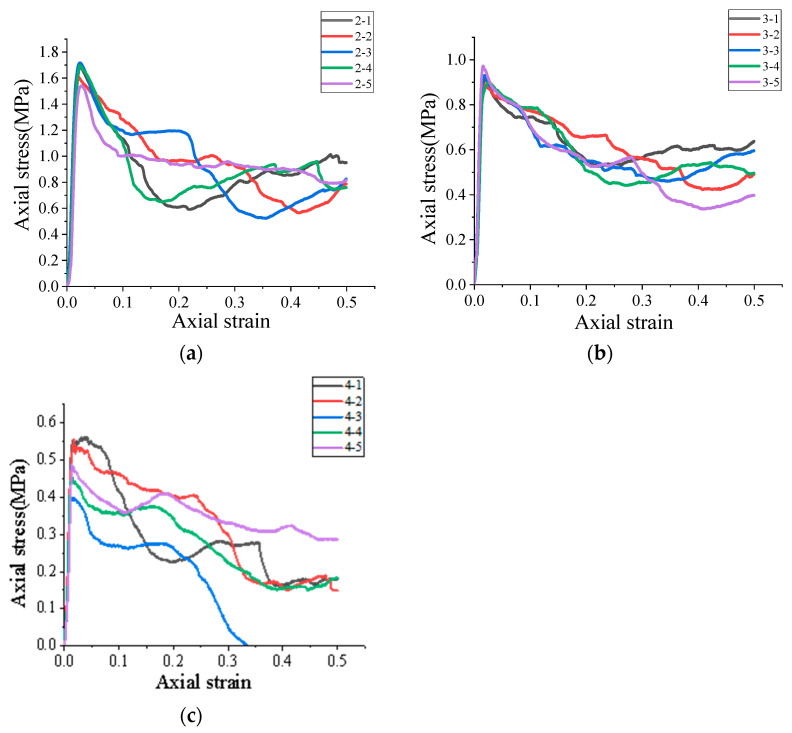
Stress–strain curve of filling material: (**a**) the water–cement ratio is 2:1; (**b**) the water–cement ratio is 3:1; (**c**) the water–cement ratio is 4:1.

**Figure 5:**
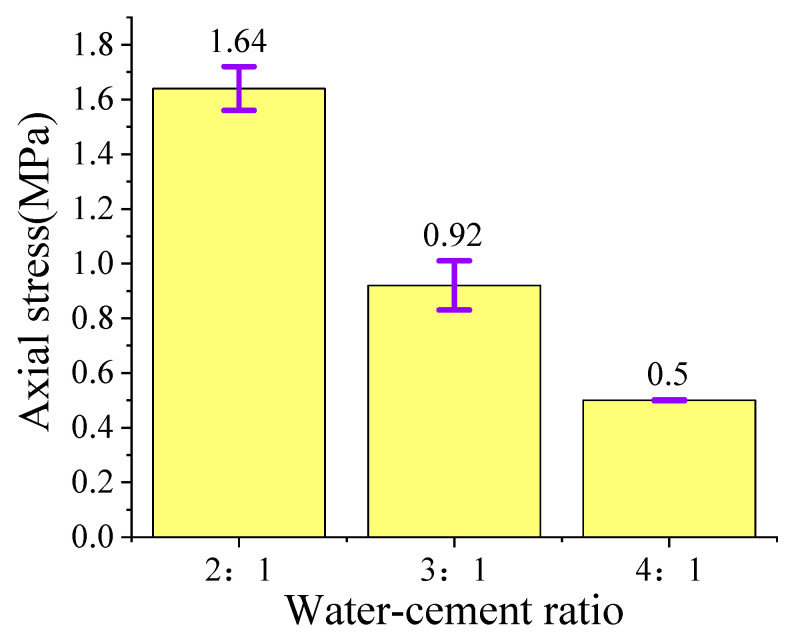
Average peak compressive strength of filling materials.

Figure 4 depicts the loading stress–strain curve of the single high-water material specimen, in which the stress is calculated according to the effective loading area of the cylinder specimen. As shown in Figure 5, the strength of the high-water material decreases with the increased water-to-cement ratio. When the water-to-cement ratio is 2:1, the maximum and the average stress reaches 1.64 MPa. However, the strength of the high-water material with the water-to-cement ratio of 3:1 is 0.92 MPa. When the water-to-cement ratio is 4:1, the compressive strength of plain material is only 0.5 MPa. Moreover, the stress–strain curve of these specimens with a higher water-to-cement ratio is generally lower than that of its counterparts. However, these specimens with a smaller water-to-cement ratio generally exhibited a worse deformation resistance. The average strain of the specimen with the water/cement ratio of 2:1 for the first failure is 4%, that is, the first failure occurs when the compression displacement is 4 mm. The failure speed of specimen 4:1 is the fastest, that is, when the compressive displacement reaches 2 mm, the first failure will occur, and then the stress will be quickly discharged. Under normal circumstances, the loading can be stopped. However, given the strong ductility of high-water materials, we choose to continue loading until the axial strain reaches 50%, which is consistent with other specimens. Further analysis shows that the three kinds of high-water materials with the same proportion will experience a rapid unloading stage after reaching the first stress peak. Afterward, they will experience a stress stabilization stage with an axial displacement approaching 10 mm. Then, the stress–strain curves of the three kinds of high-water materials with the same proportion are different for the second time. The high-water material with a water-to cement ratio of 4:1 will be rapidly unloaded after the stable stage, and some specimens cannot even be loaded to 50 mm and will completely lose their strength, while the high-water material with 2:1 and 3:1 ratios will experience the next stress growth stage. However, for the specimens with a water–cement ratio of 2:1, there are certain differences in the stress–strain evolution law of each specimen in the same group. The stress–strain curve evolution law of the high-water material with a water-to-cement ratio of 3:1 is basically the same. The possible reason for this phenomenon is the problem at the stage of grouting test piece production. The fluidity of the high-water material with a 2:1 ratio is poor after mixing and stirring for two minutes, and the quality after filling into the film is also affected. The low pumpability is not applicable to the mine filling site. Therefore, the high-water material with a water-to-cement ratio of 3:1 is used as the filling material of the basic experimental group.

**Figure 6:**
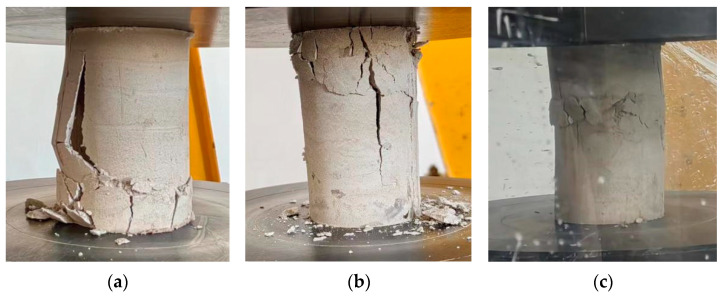
Failure modes of typical high-water material specimens: (**a**) bottom failure; (**b**) top failure; (**c**) central damage.

By observing the compression failure process of 15 high-water material specimens, we found that there was no excessive correlation between the failure mode and the water-to-cement ratio. Among the fifteen specimens, three specimens were damaged from the bottom and only one specimen was damaged from the middle, and the remaining twelve specimens were first damaged from the top. Figure 6 shows these three typical failure modes of high-water material. We believe that the top failure is a normal form of failure behavior of the specimen, because the top of the specimen is a free surface after grouting, and there is no limiting effect of the top at the top. The top strength of the formed specimen is low, and the rupture starts from here.

Figure 6a shows the typical failure mode of the specimen in the form of surface flake fall, and Figure 6b represents the failure mode of other specimens. It is apparent that several cracks developed at the top of the specimen. With the increase in pressure applied on the specimen, a main crack will appear and continue to develop until it runs through the specimen. Finally, the specimen shows the failure in the form of splitting. Figure 6c shows the only specimen with the middle failure. The specimen is separated up and down until the specimen is completely destroyed when loaded, and the top and bottom two parts will develop several cracks, but still basically maintain integrity.

### 3.2. Constrained Materials

The FRP composites used in this experiment were manufactured by Donglian Environmental Protection Technology Co., Ltd., located in Guangzhou, China. The product is known as “Glass Fiber Wound Pipe” with a specification of DN100, which were produced as per the standard HG/T21633-1991 [29] (Glass Fiber-Reinforced Plastic Pipes and Fittings). The applied FRP material is a Glass Fiber-Reinforced Composite (GFRP), and the reinforcement material is alkali-free glass fiber winding yarn. When the glass fiber is adopted as a reinforcement material, the high strength and rigidity of the composite specimens will be kept associated with a relatively lower price. The inner lining of the composite material consists of vinyl-based resin and structural benzene resin. Cobalt benzoate and methyl ethyl ketone are used as accelerators added to the resin.

**Figure 7:**
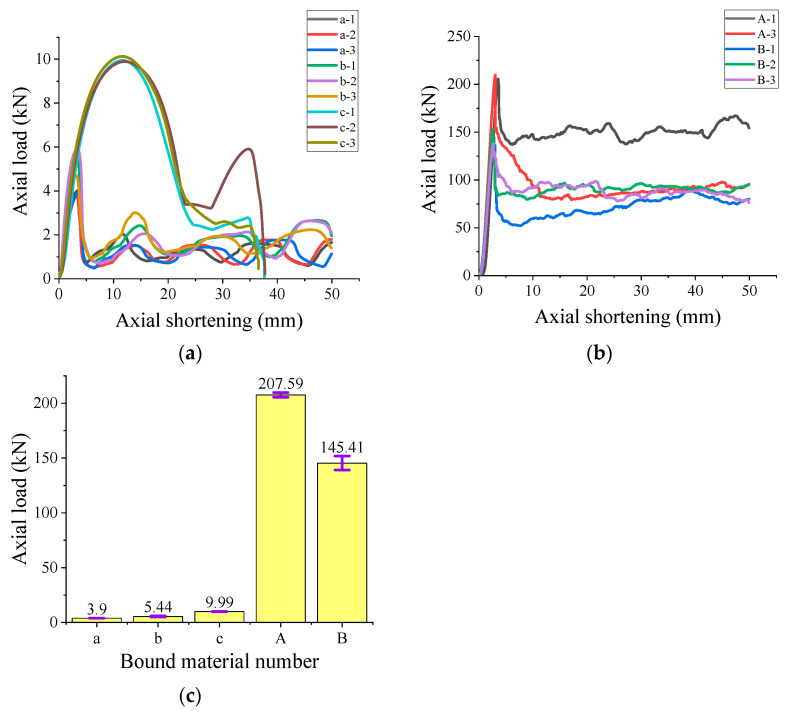
Statistical histogram of axial pressure properties and peak mean value of constrained materials: (**a**) PVC constrained; (**b**) FRP confinement; (**c**) average value of peak axial pressure of FRP pipe and PVC pipe under different parameters.

Figure 7a,b show the axial pressure property curves of the single-constrained materials. It is apparent that the PVC thickness has a great influence on its mechanical properties. For the PVC pipe numbered c, the axial shortening limit length of the material is 37 mm on average and it will fall over. That is, the axial pressure curve is basically featured with an M-shape. In detail, the axial pressure curve presents two peaks, and the peak axial pressure appears at the first peak. Although its axial deformation ability is weak, it has a high strength. As can be seen from Figure 7c, the average axial load pressure reaches 9.99 kN. For these PVC pipes numbered a and b with the same thickness and different inner diameters, the axial pressure curve is in the form of multiple peaks. Most importantly, the peak of axial pressure appears at the first peak, and the deformation capacity is stronger than PVC-c. The axial pressure shows stage growth and decay. The larger the inner diameter of the pipe, the stronger the ultimate compressive capacity. The restraint materials numbered A and B are FRP hollow tubes with thicker walls and FRP hollow tubes with thinner walls, respectively. In comparison, the ultimate compressive capacity of FRP-A is about 63 kN higher than that of the ultimate compressive capacity of FRP-B, and the curves are in the form of a single peak, and the axial compression peak will appear within the range of 3–5 mm. Different from PVC pipes, FRP tube can still maintain a stable bearing performance after the peak axial pressure, and the bearing capacity is higher. More detailed results of tested specimens are shown in Table 2.

**Figure 8:**
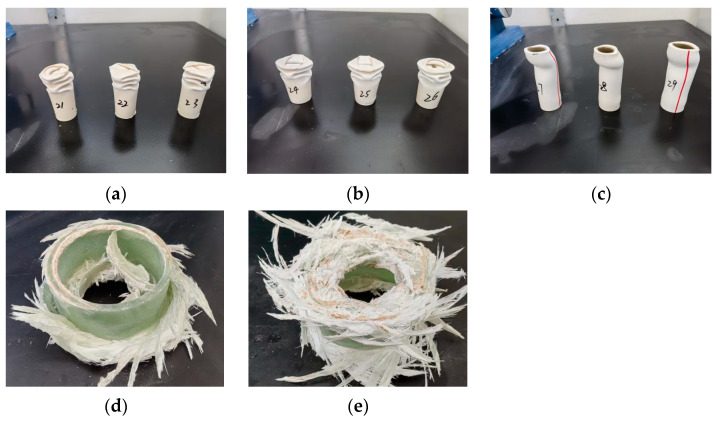
Typical failure modes of confined materials: (**a**) PVC-a; (**b**) PVC-b; (**c**) PVC-c; (**d**) FRP-A; (**e**) FRP-B. The different numbers in the figure represent sequential sample identifiers used for easy distinction during the experimental process.

As can be seen in Figure 8, the failure forms of PVC-a and PVC-b show a gradual decline and shortening, which also correspond to the emergence of multi-peak forms in the axial pressure curve. However, PVC-c has poor deformation ability, and after two significant deformations of the material, an uneven distribution of the bearing capacity on the top surface appears, which ultimately leads to the toppling of the specimen, which is the main reason why it cannot be loaded to the axial shortening amount of 50 mm. The FRP bound material begins to fail from one end, and the winding of the internal fibers ensures that the failure is steadily shortened in the axial direction. The axial pressure reaches a peak during the first failure, and then becomes stable during the stable failure stage. Of the six FRP experimental empty tubes, only one began to break both ends at the same time, which was related to the winding direction of the internal fiber during FRP processing.

### 3.3. Influence of Different Parameters on Axial Mechanical Properties of Composite Specimens

#### 3.3.1. High-Water Material Ratio

**Figure 9:**
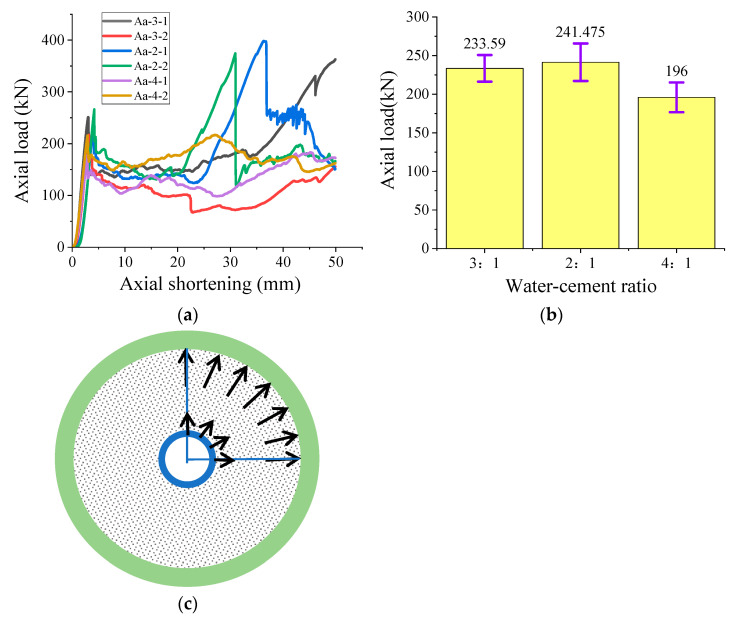
Influence of high-water material ratio on composite specimens: (**a**) axial pressure curve of composite specimens with different ratio; (**b**) the average value of peak axial pressure of composite specimens with different proportions of high-water materials; (**c**) high-water material force analysis (only 1/4 part shown, the black arrows on the inner and outer layers of the composite specimen represent the schematic representation of the reactive force of the central PVC tube on the filling material and the restraining force of the filling material on the external confining FRP tube, respectively).

Figure 9a,b show the effect of water-to-cement ratio on the axial mechanical properties of composite specimens. Similarly, for these specimens with a smaller water-to-cement ratio, the axial compressive resistance is relatively higher. This observation is consistent with the conclusion obtained in the mechanical test of a single high-water material specimen. Among them, the axial bearing performance of the composite specimen with a 4:1 water–cement ratio of high-water material is 45.475 kN worse than that of the composite specimen with a 2:1 water–cement ratio, which is related to the water–cement ratio to a certain extent. On the other hand, a large amount of water is extruded from the composite specimen with a 4:1 water–cement ratio during the compression process. Compared with the axial pressure curve of the FRP-constrained material, the trend of the constrained material basically conforms to the change law that the axial shortening amount of the combined specimen reaches the peak axial bearing pressure within 5 mm, and then it is quickly unloaded and finally reaches the stable bearing performance stage. When the axial shortening of the combined specimen reaches 50 mm, the loading automatically stops. Note that the axial pressure curve of the composite specimen with a 2:1 water–cement ratio of high-water material is different. That is, there will be a stage of rapid rise in the bearing capacity after the axial bearing capacity is stabilized. Since the experimental variable is only the proportion of high-water material, the difference caused by the water-to-cement ratio is regarded as the main factor to be considered.

The internal wall of the FRP tube subjected to the high-water sandwich material is further analyzed in this section. According to the observation obtained from the axial mechanics experiments on a single high-water material and bound material, the load-carrying capacity of the FRP tube is much higher than that of high-water sandwich material. In fact, the force between the high-water sandwich material and the FRP bound tube is a mutual force. The sandwich high-water material, FRP tube and PVC tube are stressed at the same time. When the FRP tube suffers from the axial loading, the PVC hollow tube bends inward on all sides. After the hollow volume is compacted, the internal radial deformation of the composite specimen basically ends, and most of the inward central forces interact with the sandwich high-water material, because the FRP tube has no radial deformation ability. With the further loading of the composite specimen, the outward acting force of the high-water material continues to increase and it is applied on the inner wall of the FRP tube. The mentioned action improves the bearing capacity of the FRP tube. As a result, the higher the strength of the high-water material, the higher the acting force is applied on the inner surface of the FRP tube. That is the main reason for the occurrence of the second bearing capacity peak for the specimen with a water-to-cement ratio of 2:1.

#### 3.3.2. Constrain Material Parameters

**Figure 10:**
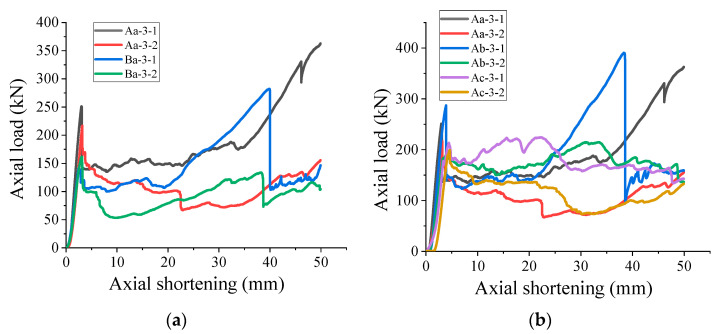
Pressure curves of combined specimens with different constrained material parameters: (**a**) different FRP tube thicknesses; (**b**) different PVC pipe parameters.

**Figure 11:**
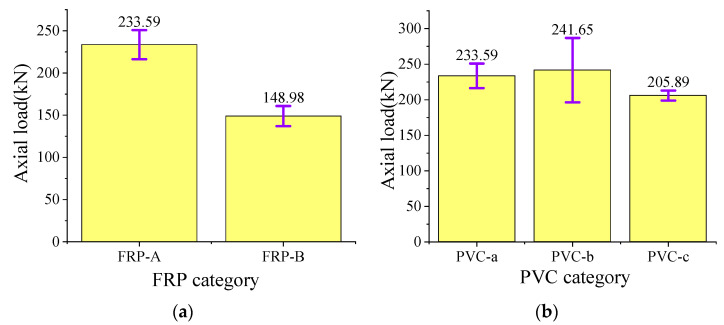
Histogram of average peak pressure of specimens with different constrained material parameters: (**a**) different FRP tube thickness; (**b**) different PVC pipe parameters.

According to the analysis of Figure 10a and Figure 11a, the thickness of the FRP-restrained tube has the most obvious effect on the axial bearing capacity of the composite specimen. The thicker the FRP tube, the stronger the axial bearing capacity of the composite specimen. The load-bearing capacity of the composite specimens (e.g., FRP-A) is 35.8% higher than that of the FRP-B specimens. It is observed from Figure 10b and Figure 11b that the overall axial carrying capacity of the composite specimen is directly proportional to the inner diameter of the PVC pipe and inversely proportional to the thickness of the PVC pipe. This observation is inconsistent with the above analysis that the PVC pipe with a larger inner diameter and thicker thickness generally had a larger axial carrying capacity.

**Figure 12:**
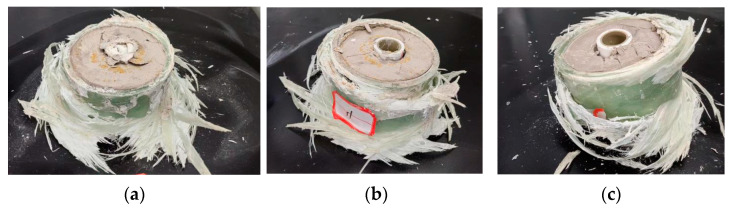
Failure mode of a built-in PVC pipe in typical composite specimens: (**a**) The built-in PVC pipe is PVC-a; (**b**) the built-in PVC pipe is PVC-b; (**c**) the built-in PVC pipe is PVC-c.

The further analysis of the failure mode of a typical PVC pipe in Figure 12 shows that PVC-c PVC pipe has large thickness and poor deformation ability. During the loading process, the damage degree is gradually inconsistent with other parts of the composite specimen, which is consistent with the above analysis result of the failure mode of the single PVC pipe. The axial deformation ability is poor and eventually evolves into radial inclination, which damages the integrity of the sandwich high-water filling material and ultimately leads to the weakening of the overall axial bearing capacity of the combined specimen. This is significantly different from the failure mode of PVC-a pipe under axial compression deformation without radial deformation as shown in Figure 12a.

## 4. Discussion

**Figure 13:**
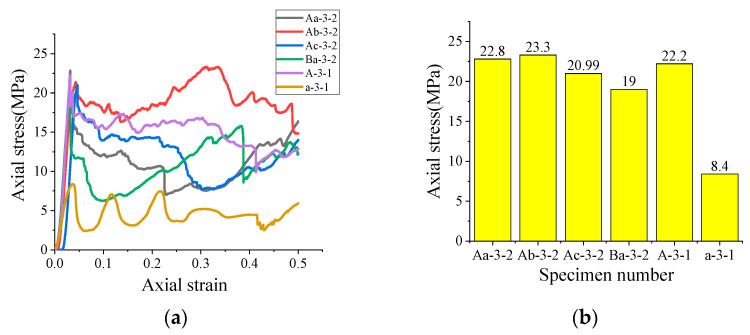
Stress–strain curve and average stress peak value of typical specimens: (**a**) stress–strain curve of typical specimens; (**b**) average value of peak stress of typical specimens.

Under the condition that the water–cement ratio of the optimal high-water material screened in the above section is 3:1, in order to further discuss the relationship between the overall axial bearing capacity of the combined specimen and the axial bearing capacity of each part of the combined specimen, the axial mechanical property data are further processed, and several groups of typical stress–strain curves of the combined specimen are obtained, as shown in Figure 13. It can be seen from the analysis that only the thickness of FRP tube has the greatest influence on the overall compressive strength of the composite specimen among all the parameters of the constrained materials, which is consistent with the conclusion obtained from the experiment on the mechanical properties of the single constrained material in the above section. Except for the composite specimens using PVC-c, the strength of the composite specimens with a certain hollow ratio is greater than that of the solid FRP–high-water material composite specimens. This indicates that the hollow condition is conducive to improving the overall compressive strength of the composite specimen. In addition, it can be found that the compressive strength of the composite specimen with a large hollow rate is higher than that of the small-hole heart-rate composite specimen; the compressive strength of the composite specimen with a smaller PVC pipe thickness is higher; and the compressive strength of the composite specimen with a larger FRP thickness is higher. Therefore, in summary, PVC-c (inner diameter: 29 mm; external diameter: 32 mm) is preferred. FRP-A (internal diameter: 100 mm, external diameter: 112 mm) and a high-water material with a water/cement ratio of 3:1 were used as the combination of specimens with the best axial mechanical properties.

**Figure 14:**
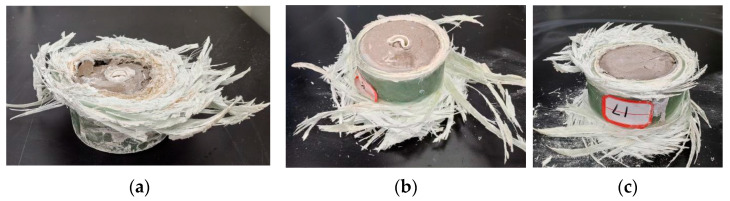
Failure forms of typical specimens: (**a**) top failure mode; (**b**) bottom failure mode; (**c**) simultaneous top and bottom failure mode.

In Figure 14, three groups of typical failure modes of the combined specimens were selected. Figure 14a shows failure at the top of the specimen, Figure 14b shows failure at the bottom of the specimen, and Figure 14c shows simultaneous failure at the top and bottom of the specimen. Statistics were made on the failure modes of 16 groups of the combined specimens. There was only one specimen that failed at the same time at the top and bottom, and the failure mode was not significantly related to the PVC pipe type and high-water material ratio inside the composite specimen, but only related to the nature of FRP itself, and the initial estimate was related to the starting position and winding direction of the internal fiber entangling when the FRP pipe was in operation. Table 3 shows the statistics of key data.

**Figure 15:**
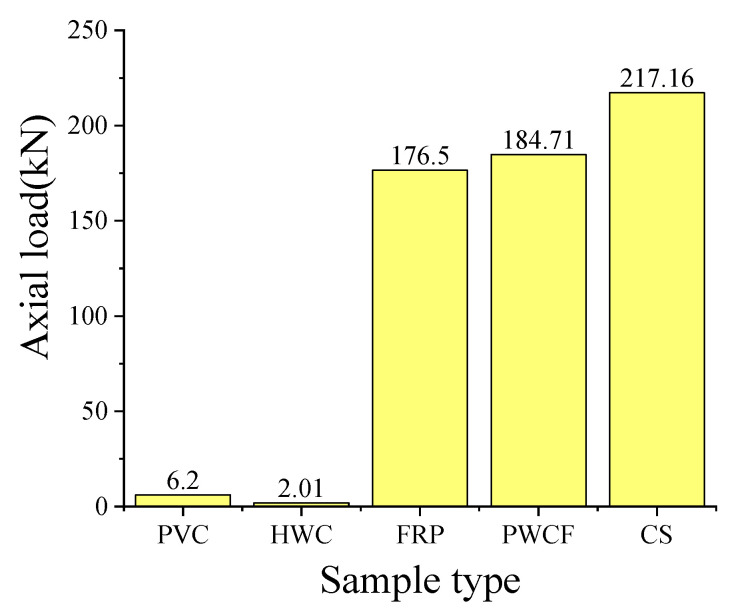
Histogram of the average value of the peak axial pressure of each part of the constrained material and the combined specimen. (PVC: Polyvinyl chloride, HWC: high-water cement, FRP: fiber-reinforced polymer, PWCF: the sum of the strengths of each component material, CS: composite specimen).

For these composite specimens, PVC-c is tilted to one side due to the thickness of the internal PVC pipe during compression. This typical failure mode will damage the overall integrity and degrades the bearing performance of the composite specimen. In particular, these composite specimens with a higher water-to-cement ratio (i.e., 4:1) generally exhibited obvious water squeezes during the loading process. There is no doubt that the integrity of the specimen deterioration, and the overall bearing performance of the composite specimen decreases. Moreover, the experiment basically shows that the axial bearing capacity of the combined specimen as a whole is greater than the sum of the individual bearing capacity of each part of the combined specimen. Figure 15 shows the average value of the peak axial pressure of each part of the constrained material and the combined specimen. It can be seen that the bearing capacity of the combined specimen is 14.9% higher than the sum of the bearing capacity of all components. This is believed to be the other evidence to support that the bearing capacity of the combined specimen is significantly improved. 

For these specimens made of three different materials, the confining action will enhance the overall mechanical performance of the specimen under the uniaxial loading. When the combination forms and filling material types are different [30,31,32,33,34], the combination forms will also vary. A good example is that the double-wall hollow structure [30] of the FRP pipe directly restrained the PVC pipe wrapped in filling material [31], and the FRP pipe indirectly restrained the PVC pipe with internal filling material [32]. That is, both the FRP pipe and PVC pipe presented sandwich packing foam hairspray and other flexible support, while the FRP pipe strip restrained the PVC pipe packing and filling materials [33]. However, the main filling material of these mentioned composite structures are concrete. With the development of coal slag separation technology, coal gangue-cemented grouting filler has also been applied [13]. Similar to the structural form presented in this research, double-wall hollow structures with PVC pipes embedded in the inner wall and filled with high-water materials are not well investigated. The findings by Goaiz, H, and Dinesh G on PVC pipe double-wall hollow structures can be referred to in [34,35]. Their investigation verified that the concrete strength has been enhanced by 87% with the active confinement provided by the exterior container. Although the inner tube reduces the proportion of filling materials, their test results still show that the strength of the combined specimens has not decreased, which is consistent with the research results in this paper. Secondly, with the addition of PVC pipe as the inner pipe, the ductility and deformation resistance of the composite specimen are significantly improved compared with that of the non-hollow composite specimen, which is also consistent with the failure mode analysis of the composite specimen in this paper [34,35].

## 5. Conclusions

This paper presents an innovative structural form for underground mines, the exterior container of which is the double-wall column made of FRP and PVC, whereas the high-water material is filled in between. Compared to the three components, the load-bearing capacity of the composite column is increased by 14.9% under the interaction between FRP pipe, high-water material, and PVC. In addition, the composite column exhibited a good post-failure load-bearing capacity. 

The axial compressive stress–strain curves of these double-wall hollow columns are classified into three groups as per the shape of the curves. When other parameters are all the same, the strength of the high-water material is believed to be proportional to the axial mechanics of the double-wall hollow column. That is, these composite columns with a greater strength of high-water material will exhibit a superior axial stress. With the increase in confinement provided by the thicker FRP tube, both the loading-carrying capacity and the deformation ability of the composite columns will be enhanced. Different from FRP tube with high strength-to-weight ratio, the effect of PVC thickness is not obvious. 

In general, these specimens with a PVC-c (inner diameter: 29 mm, outer diameter: 32 mm), FRP-A (inner diameter: 100 mm, outer diameter: 112 mm), and high-water material with a water-to-cement ratio of 3:1 is the ideal structural form for evaluating the axial mechanical properties.

## Figures and Tables

**Table 1:** Details of the test specimens.

Series	Group	Specimen Code	Confinement Material				Water-to-Cement Ratio	Number of Specimens
			Type	Thickness of FRP (mm)	Thickness of PVC (mm)	Inside Diameter of PVC (mm)		
I	Aa-2	Aa-2-1,2	PVC + FRP	6	1.5	22	2:1	2
	Aa-3	Aa-3-1,2	PVC + FRP	6	1.5	22	3:1	2
	Aa-4	Aa-4-1,2	PVC + FRP	6	1.5	22	4:1	2
II	Aa-3	Aa-3-1,2	PVC + FRP	6	1.5	22	3:1	2
	Ab-3	Ab-3-1,2	PVC + FRP	6	1.5	29	3:1	2
	Ac-3	Ac-3-1,2	PVC + FRP	6	5	22	3:1	2
III	Aa-3	Aa-3-1,2	PVC + FRP	6	1.5	22	3:1	2
	Ba-3	Ba-3-1,2	PVC + FRP	3	1.5	22	3:1	2
IV	A-3	A-3-1,2	FRP	6			3:1	2
	a-3	a-3-1,2	PVC		1.5	22	3:1	2
V	A	A-1,2,3	FRP	6				3
	B	B-1,2,3	FRP	3				3
	a	a-1,2,3	PVC		1.5	22		3
	b	b-1,2,3	PVC		1.5	29		3
	c	c-1,2,3	PVC		5	22		3
VI	1	1-1,2,3,4,5					2:1	5
	2	2-1,2,3,4,5					3:1	5
	3	3-1,2,3,4,5					4:1	5

**Table 2:** Constrained material key parameters.

Constrained Material Type	Specimen Code	Peak Axial Pressure (kN)	Peak Mean (kN)	Strain	Stress (MPa)	Mean Stress
FRP	A-1	205.4	207.6	50	102.8	103.9
	A-2	null		null	null	
	A-3	209.8		50	105.0	
	B-1	145.4	145.4	50	149.7	149.8
	B-2	153.2		50	157.8	
	B-3	137.6		50	141.8	
PVC	a-1	4.0	3.9	50	36.2	29.9
	a-2	3.8		50	26.4	
	a-3	3.9		50	27.1	
	b-1	5.7	5.4	50	39.8	37.9
	b-2	5.9		50	41.2	
	b-3	4.7		50	32.8	
	c-1	9.9	9.9	37.9	23.5	23.7
	c-2	9.9		38	23.5	
	c-3	10.1		36.7	24.0	

**Table 3:** Key data.

Series	Specimen	$P_{water-cement}$ (kN)	$P_{pvc+frp}$ (kN)	$P_{Composite-specimen}$ (kN)	$P_{water-cemrnt+pvc+frp}$ (kN)	$\frac{P_{Composite-specimen}}{P_{water-cement}+P_{pvc+frp}}$	Mean Value
I	Aa-2-1 Aa-2-2	3.24 3.24	211.5 211.5	217.2 265.7	214.74 214.74	1.01 1.24	1.125
	Aa-3-1 Aa-3-2	1.8 1.8	211.5 211.5	250.8 216.4	213.3 213.3	1.18 1.01	1.095
	Aa-4-1 Aa-4-2	1 1	211.5 211.5	176.8 216.3	212.5 212.5	0.83 1.02	0.925
II	Aa-3-1 Aa-3-2	1.8 1.8	211.5 211.5	250.8 216.4	229.5 229.5	1.18 1.01	1.095
	Ab-3-1 Ab-3-2	1.8 1.8	213 213	286.9 196.4	214.8 214.8	1.34 0.91	1.125
	Ac-3-1 Ac-3-2	1.8 1.8	217.5 217.5	213 198.8	219.3 219.3	0.97 0.91	0.94
III	Aa-3-1 Aa-3-2	1.8 1.8	211.5 211.5	250.8 216.4	213.3 213.3	1.18 1.01	1.095
	Ba-3-1 Ba-3-2	1.8 1.8	149.3 149.3	141 160.9	151.1 151.1	0.93 1.06	0.995

## Data Availability

Not applicable.
